# The “LIME Wedge”: A Technical Report on a 3D-Printed Radiologic Reference Device for Differentiating Button Battery and Coin Ingestion

**DOI:** 10.7759/cureus.106545

**Published:** 2026-04-06

**Authors:** Peyton J Ware, Carter Ware, Amanda H Bjornstad, Heather M Kuntz, Timothy P Young

**Affiliations:** 1 Laboratory for Innovations in Medical Education, Loma Linda University School of Medicine, Loma Linda, USA; 2 Radiology, Texas Tech University Health Sciences Center El Paso, El Paso, USA; 3 Emergency Medicine, Loma Linda University Medical Center, Loma Linda, USA; 4 Pediatric Emergency Medicine, Loma Linda University Medical Center, Loma Linda, USA

**Keywords:** 3d-printing, button battery, coins, foreign body, radiologic reference

## Abstract

Button battery ingestions are time-sensitive emergencies that require prompt identification and removal. Button batteries can be mistaken for coins, which can lead to dangerous delays in management. We designed the “LIME Wedge” radiologic reference device to hold four coins and one 20 mm button battery. We used a radiologic phantom to simulate button battery and coin ingestions on anterior and lateral chest X-ray (CXR) images. This series of images demonstrates how our tool can be used to help visually differentiate esophageal button batteries from coins. The LIME Wedge is an inexpensive, 3D-printed, radiologic tool that provides physicians with reference examples of various coins and a button battery in two radiologic planes. This radiologic tool is accessible for free download in the appendices.

## Introduction

The diagnosis of ingested foreign bodies (FB) relies on patient history and radiographic images. Coins are the most commonly ingested foreign body in the pediatric population [1]. Button batteries (BBs) are similar-looking, circular FBs that are significantly more dangerous. BBs cause rapid injury from a pH imbalance that occurs when the anode is constantly exposed to a moist environment; the resulting reaction along the gastrointestinal mucosa leads to liquefactive necrosis that can result in esophageal burns, esophageal perforation, vascular damage, transesophageal fistulas, and strictures [2,3]. Characteristic radiographic evidence helps to differentiate BB from other circular FBs: two concentric rings known as the “halo sign” and a beveled or rounded edge known as the “step-off sign” [4,5]. Radiologists are only 80.5% accurate in distinguishing a button battery from a coin [6]. In the largest case series of button battery ingestions in children, 7 of 13 deaths involved a missed diagnosis by a healthcare provider [7]. While patient history may suggest coin ingestion, Frumkin and Lanker urge providers to “look again” at imaging to ensure a BB ingestion is not missed [8].

BBs are commonly found in household objects, including car keys, hearing aids, toys, watches, and air tags. The American Academy of Pediatrics recommends keeping BBs out of children’s reach to prevent injury [9]. However, the ubiquitous nature of BBs makes this difficult to accomplish. The hidden danger of BB integration into daily life led to governmental, manufacturing, and clinical coordination within the “Button Battery Task Force” to innovate novel approaches to ingestion prevention [5]. Recent developments in BB manufacturing include dyes that stain the oral mucosa upon ingestion and bitter-tasting coatings to discourage children from swallowing a BB [10,11]. Nonetheless, these coatings are not universal among manufacturers.

BBs must be promptly identified and removed to prevent injury progression [12]. Definitive recognition on X-ray may be difficult because BBs can appear radiographically similar to coins. This may cause a delay in definitive management of a BB ingestion. All US coins have a ridge that can be misinterpreted as the double concentric ring seen with BBs [13]. Our 3D-printed button battery-coin holder serves as a radiologic reference that allows physicians to compare ingested FBs with various coins and a BB on X-ray. This may help them identify and appropriately manage a child with a BB ingestion.

## Technical report

**Figure 1:**
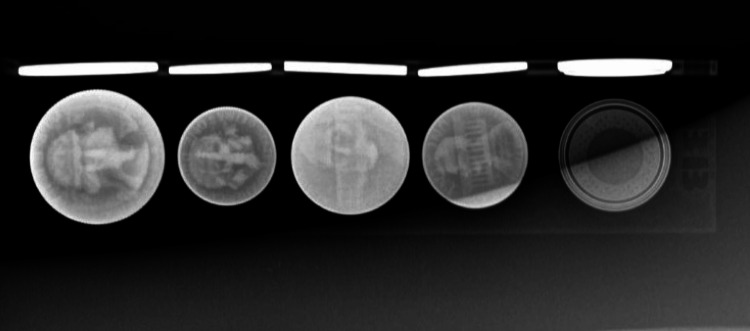
X-ray image of the LIME Wedge coin and button battery holder Objects in the holder from left to right: quarter, dime, nickel, penny, and button battery.

**Figure 2:**
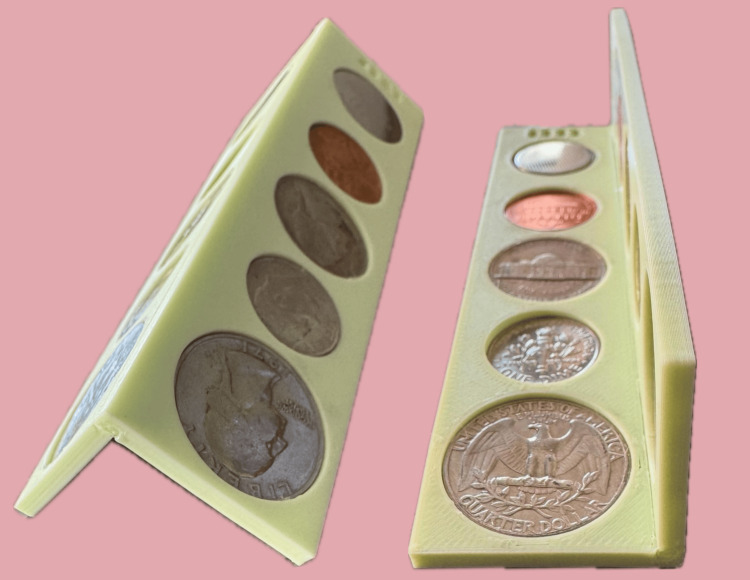
The final LIME Wedge with coin and button batteries in place

We designed the “LIME Wedge” radiologic reference device to hold four coins (penny, nickel, dime, and quarter) and one 20 mm button battery (CR2025, CR2032, or CR2016) (Figure 1). We modeled the radiologic reference with the freely available Tinkercad (Autodesk, San Francisco, California) [14]. A stereolithography (STL) file of the model was converted to a printer-readable file, also known as sliced, using the open-source Prusa Slicer (Prusa Research, Prague, Czech Republic) [15]. We printed the device in two separate parts with a Prusa Mini+ (Prusa Research, Prague, Czech Republic) [16]. We used matte polylactic acid (PLA) filament (Elegoo, Shenzhen, China) [17] at a nozzle temperature of 210-215° C with 15% infill. The coins and button battery press-fit into the mold. The LIME Wedge pieces fit to form a right angle that can be glued together with cyanoacrylate glue (Figure 2). To demonstrate the utility of the LIME Wedge across printers and PLA from different brands, we also successfully printed the LIME Wedge on a Bambu Lab P1S with a different PLA filament brand after the STL file was sliced by the open-source BambuStudio (Bambu Lab, Shenzhen, China) [18,19].

**Figure 3:**
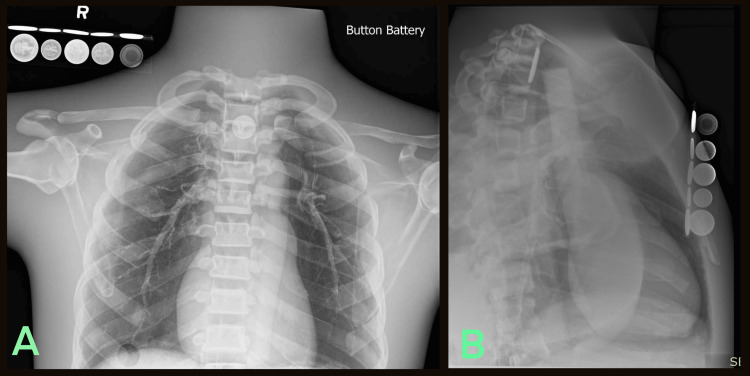
Radiologic phantom images of simulated button battery ingestion accompanied by the LIME Wedge radiographic reference (A) Anteroposterior and (B) Lateral.

**Figure 4:**
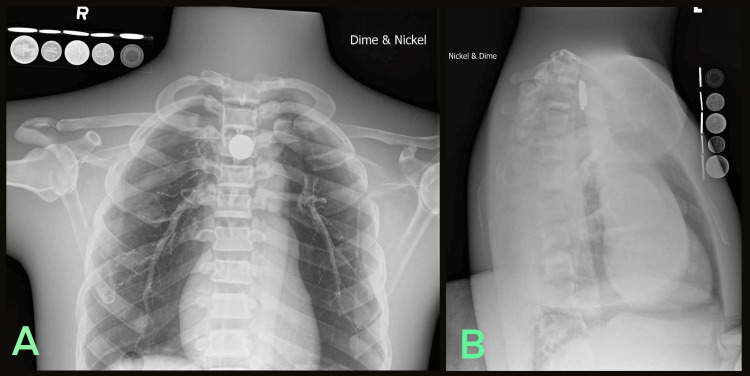
Radiologic phantom images of simulated dime and nickel coin stack ingestion accompanied by the LIME Wedge radiographic reference. (A) Anteroposterior and (B) Lateral.

**Figure 5:**
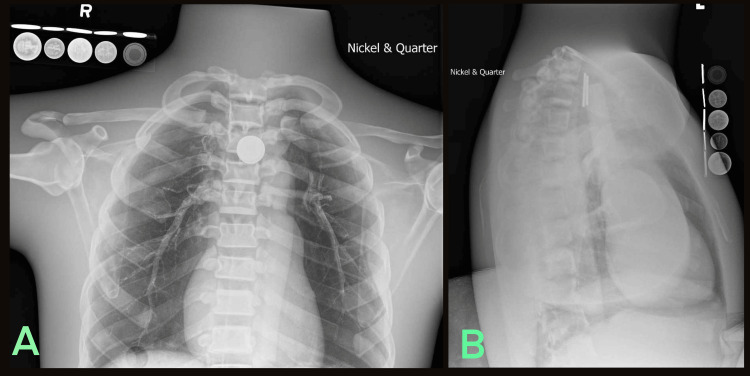
Radiologic phantom images of simulated nickel and quarter coin stack ingestion accompanied by the LIME Wedge radiographic reference (A) Anteroposterior and (B) Lateral.

**Figure 6:**
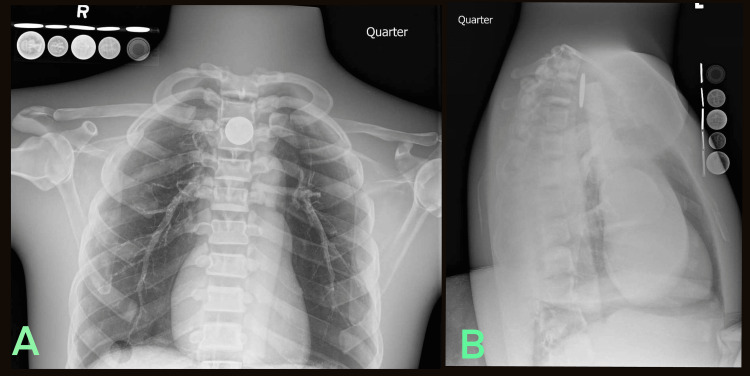
Radiologic phantom images of simulated quarter ingestion accompanied by the LIME Wedge radiographic reference (A) Anteroposterior and (B) Lateral.

**Figure 7:**
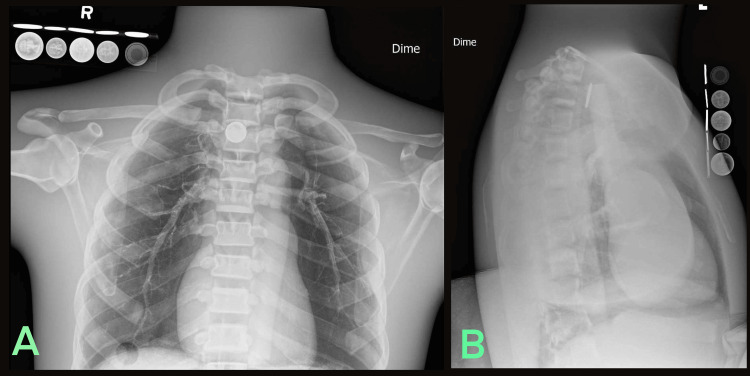
Radiologic phantom images of simulated dime ingestion accompanied by the LIME Wedge radiographic reference (A) Anteroposterior and (B) Lateral.

**Figure 8:**
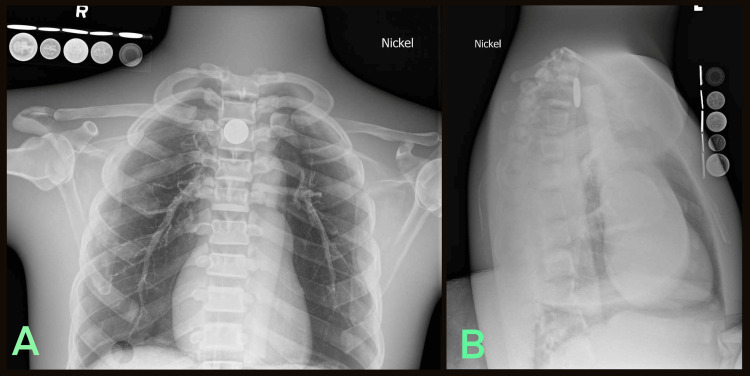
Radiologic phantom images of simulated nickel ingestion accompanied by the LIME Wedge radiographic reference (A) Anteroposterior and (B) Lateral.

**Figure 9:**
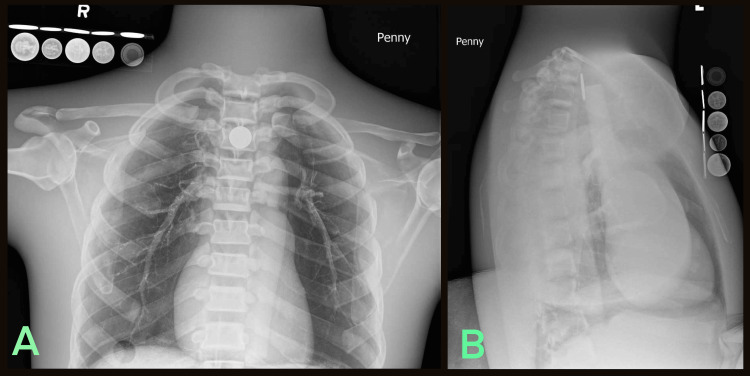
Radiologic phantom images of simulated penny ingestion accompanied by the LIME Wedge radiographic reference (A) Anteroposterior and (B) Lateral.

We used a radiologic phantom to simulate BB and coin ingestions on anterior and lateral CXR images (Figures 3-9). The series of images includes simulated ingestions of a BB, dime, and nickel coin stack, a nickel and quarter coin stack, a quarter, dime, nickel, and penny. A print-ready STL and editable version of the LIME Wedge are provided in the appendix.

We used less than 0.50 USD worth of filament to print the LIME wedge and the print time was about 1.5 hours on the Prusa printer and 0.5 hours on the Bambu printer.

## Discussion

We created an inexpensive radiologic tool that contains the halo and step-off signs to help visually differentiate esophageal button batteries from coins. The device also acts as a physical reminder to include the “can't-miss” diagnosis of button battery ingestion on the differential diagnosis. At our institution, emergency physicians store the tool in their work area in the emergency department, as they would other clinical aids (e.g., ultrasound, tonometer, etc.). When a patient presents with concern for an ingestion, the physician can provide this tool to the radiology technician to place next to the patient during their initial X-ray. In this way, its use does not increase radiation exposure. Its presence can also serve as an auxiliary prompt to the interpreting radiologist for the clinical context. 

**Figure 10:**
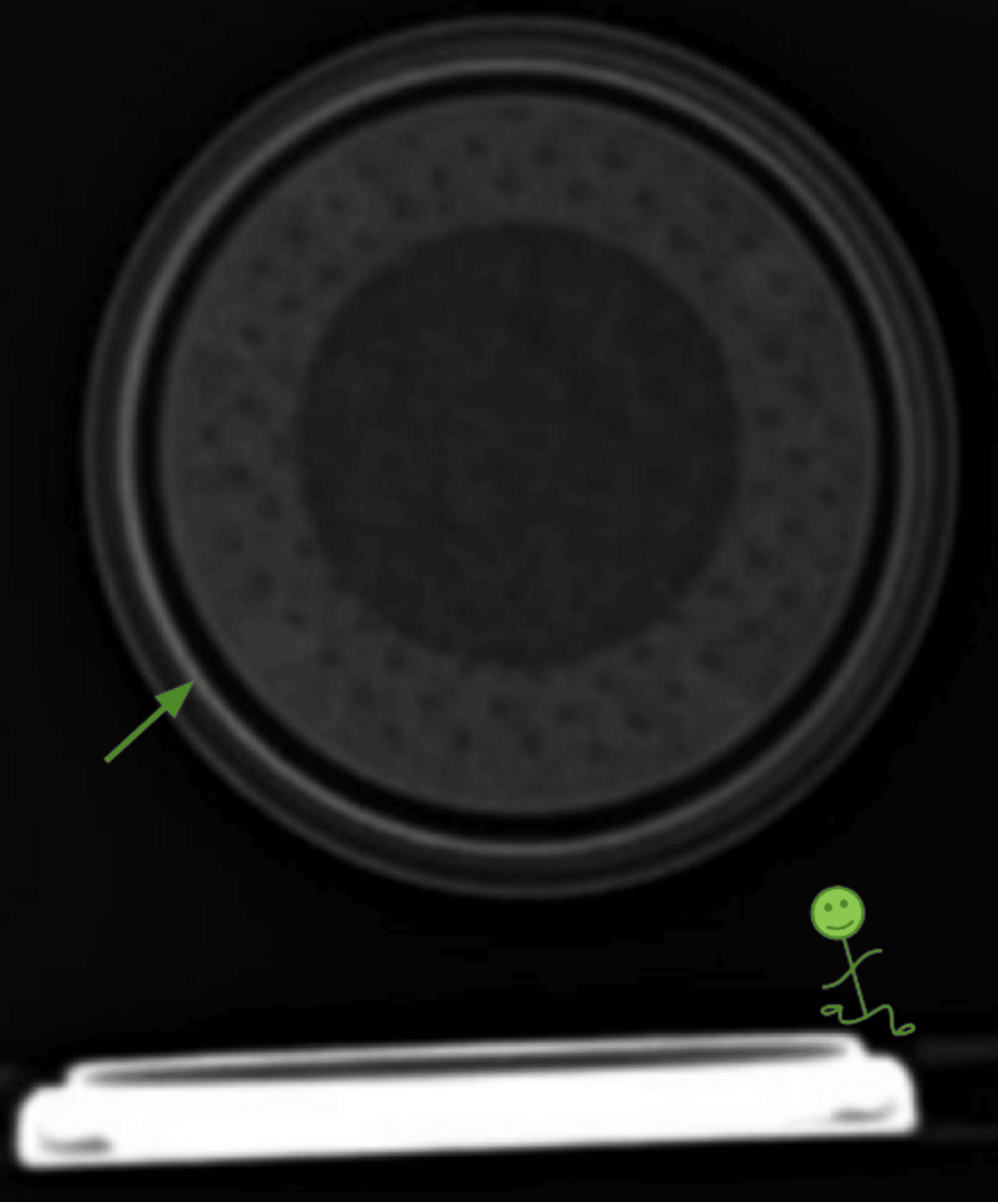
Halo sign and step-off sign seen within LIME Wedge Halo sign (arrow) and step-off sign (stick figure) seen within LIME Wedge.

Button batteries come in many sizes. We chose the 20 millimeter button battery because it is associated with most complications [7]. We chose not to include multiple sizes because we did not want to give the impression that we were including the full breadth of potential sizes. The size of an X-ray projection also varies depending on the distance from the plate. Button battery ingestions are time-sensitive emergencies. While the halo and step-off signs (Figure 10) are most associated with BBs, coin stacks can also present with these signs [20,21]. Mistaking a BB for a coin stack ingestion can result in devastating injury if the removal of the corrosive FB is delayed. Our tool can also help visually distinguish a button battery from a coin stack.

## Conclusions

The LIME Wedge is an inexpensive, 3D-printed, radiologic tool that provides physicians with reference examples of various coins and a BB in two radiologic planes. The examples appear directly on the radiologic image and can help clinicians identify flat, round esophageal foreign bodies. Our in vitro images display a catalog of simulated ingestions and visually demonstrate the LIME Wedge’s utility in differentiating metallic foreign bodies. The tool may be useful at other institutions as well. Future studies could evaluate the LIME wedge’s effectiveness in increasing radiographic diagnostic accuracy. The STL file for the LIME Wedge is accessible for free download in the appendices.

## Appendices

https://tinyurl.com/lime-wedge

https://tinyurl.com/limewedgetinkercad
